# Supplementary material for: Study of miRNA and lymphocyte subsets as potential biomarkers for the diagnosis and prognosis of gastric cancer
Source: PeerJ. 2024 Jan 19;12:e16660. doi: 10.7717/peerj.16660 (PMC10802158; doi:10.7717/peerj.16660)
Supplement: Supplemental Information 20 [file peerj-12-16660-s020.zip › Sequence Data/Description.docx]

分析要求：

1．1）取交集的时候下调也要；2）差异的标准可以放宽到1.5倍，最好可筛选的miRNA达到10个左右。

1. 可以的目的miRNA为miR-27a-3p，目的信号通路为Wnt
2. 差异基因的Venn图
3. 靶基因预测部分数据缺失，更新

文件说明：

1. Prediction-FC2/文件夹为之前交付筛选9样本预测（prediction文件夹缺失数据内容替换）
2. DEG/目录为取（P0.05，FC1.5）差异标准绘制的各组热图及火山图结果，其中DEG-edgeR-FCPQ.xlsx为该标准下筛选出的差异基因表格，DEG-edgeR.xlsx为总表。

Ps：降低阈值后，miR-27a-3p亦仅在C_vs_H组显著差异。

1. 根据差异的miRNA进行后续预测，所用软件为miranda（参数提高至-en 30），结果存放prediction-miranda/目录，实验验证结果存放validated/目录。
2. Function/：根据靶基因预测及实验验证结果做功能富集，各分组结果分别存放对应目录。

Ps：prediction/目录下，Wnt通路在三分组中均显著，但Y_H组Wnt通路未处于前十（没有体现在KEGG_barplot.pdf图，如需要可以添加进图中）。

validated/部分，C_Y组wnt通路不显著，另两组wnt通路显著但未处于前十。

1. heatmap-*.pdf : 为修改差异标准后独有上调和添加下调的两幅热图，绘图数据为heatmap.xlsx
2. venn.pdf : 三分组各自差异基因的数目，具体基因见venn.txt。

Analysis requirements：

1．1) When taking the intersection, it should be lowered; 2) The standard of difference can be relaxed to 1.5 times, and it is best to have about 10 miRNAs that can be screened.

1. The target miRNA that can be used is miR-27a-3p, and the target signal path is Wnt

2. Venn diagram of differential genes

3. Part of the target gene prediction data is missing, updated

File description：

1. The Prediction-FC2/folder is the prediction of the 9 samples previously delivered (the prediction folder is replaced by the missing data content)

2. The DEG/table of contents is the results of each group of heat maps and volcano maps drawn by the difference standard (P0.05, FC1.5), of which DEG-edgeR-FCPQ.xlsx is a table of differential genes selected under this standard, DEG-edgeR.xlsx is the summary table.

Ps: After lowering the threshold value, miR-27a-3p is only significantly different in the C_vs_H group.

3. For subsequent prediction based on the different miRNAs, the software used is miranda (the parameter is increased to -en 30), the results are stored in the prediction-miranda/directory, and the experimental verification results are stored in the verified/directory.

4. Function/: Function enrichment is done based on the target gene prediction and experimental verification results, and the results of each group are stored in the corresponding directory.

Ps: In the prediction/directory, the Wnt pathway is significant in all three groups, but the Wnt pathway in the Y_H group is not in the top ten (not reflected in KEGG_barplot.pdf diagram, you can add it to the diagram if necessary).

In the verified/part, the wnt pathway in the C_Y group was not significant, and the other two groups of wnt pathways were significant but not in the top ten.

5. heatmap-*.pdf: It is two heat maps that can be raised and lowered after modifying the difference standard. The drawing data is heatmap.xlsx

6. venn.pdf: The number of different genes in each of the three groups, see venn for specific genes.txt.
