# Supplementary material for: Study of miRNA and lymphocyte subsets as potential biomarkers for the diagnosis and prognosis of gastric cancer
Source: PeerJ. 2024 Jan 19;12:e16660. doi: 10.7717/peerj.16660 (PMC10802158; doi:10.7717/peerj.16660)
Supplement: Supplemental Information 20 [file peerj-12-16660-s020.zip › Sequence Data/Function/validated/C_VS_H/KEGG_Pathway.barplot.pdf]

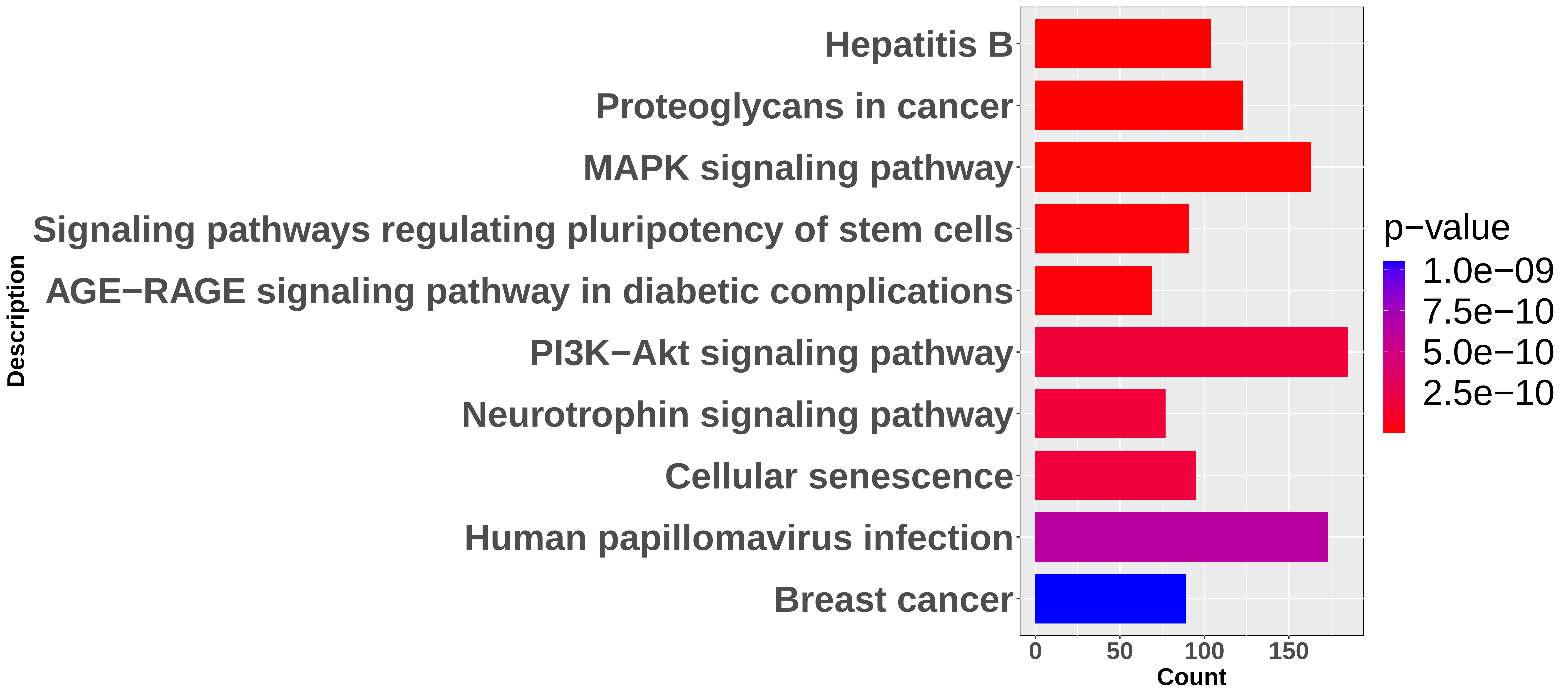
Hepatitis B
Proteoglycans in cancer
MAPK signaling pathway
Signaling pathways regulating pluripotency of stem cells
AGE−RAGE signaling pathway in diabetic complications
PI3K−Akt signaling pathway
Neurotrophin signaling pathway
Cellular senescence
Human papillomavirus infection
Breast cancer
Description
0
50
100
150
Count
p−value
1.0e−09
7.5e−10
5.0e−10
2.5e−10
