# Supplementary figures and images for: Study of miRNA and lymphocyte subsets as potential biomarkers for the diagnosis and prognosis of gastric cancer
Source: PeerJ. 2024 Jan 19;12:e16660. doi: 10.7717/peerj.16660 (PMC10802158; doi:10.7717/peerj.16660)

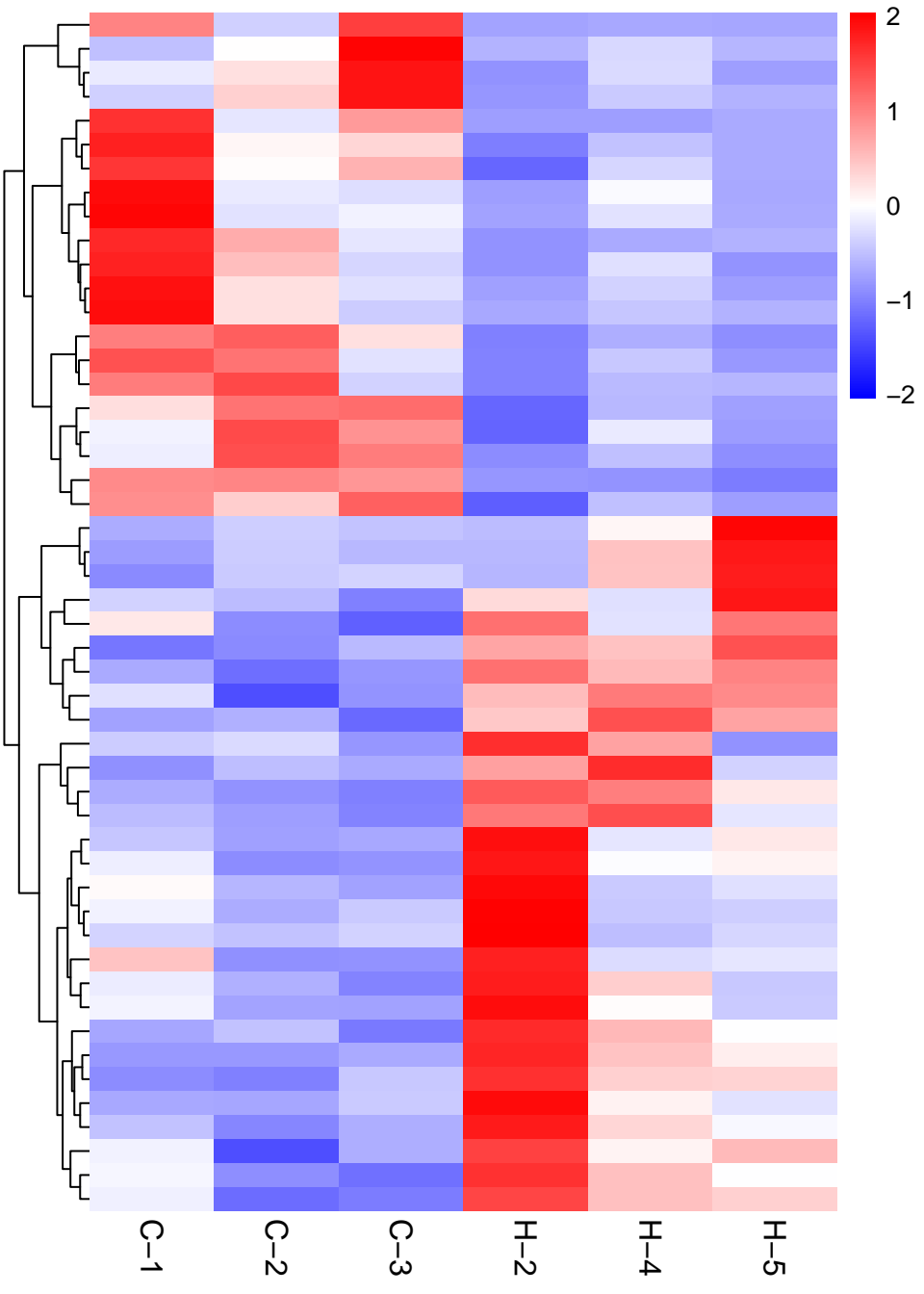

Supplement: Supplemental Information 20 [file peerj-12-16660-s020.zip › Sequence Data/DEG/C_VS_H/heatmap.pdf]

MA plot of DEG

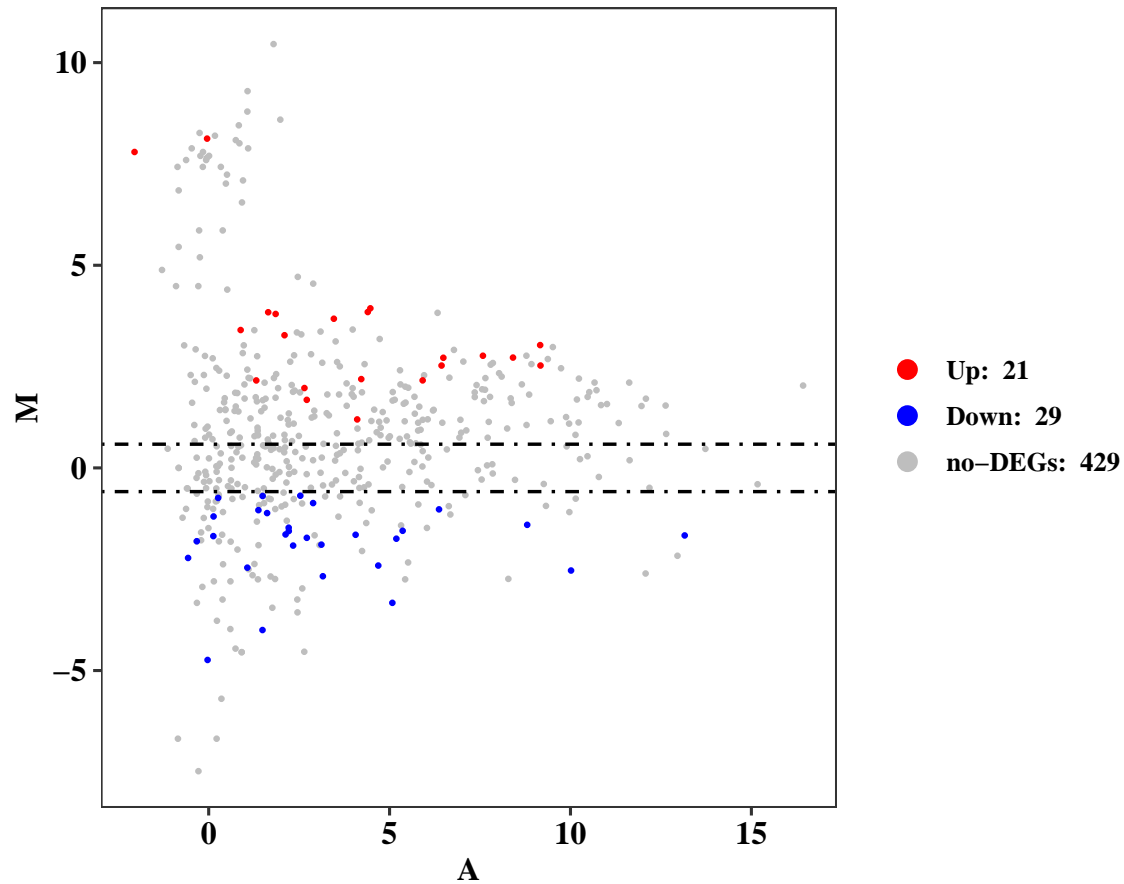

Supplement: Supplemental Information 20 [file peerj-12-16660-s020.zip › Sequence Data/DEG/C_VS_H/MA-plot.pdf]

Scatter plot of DEG

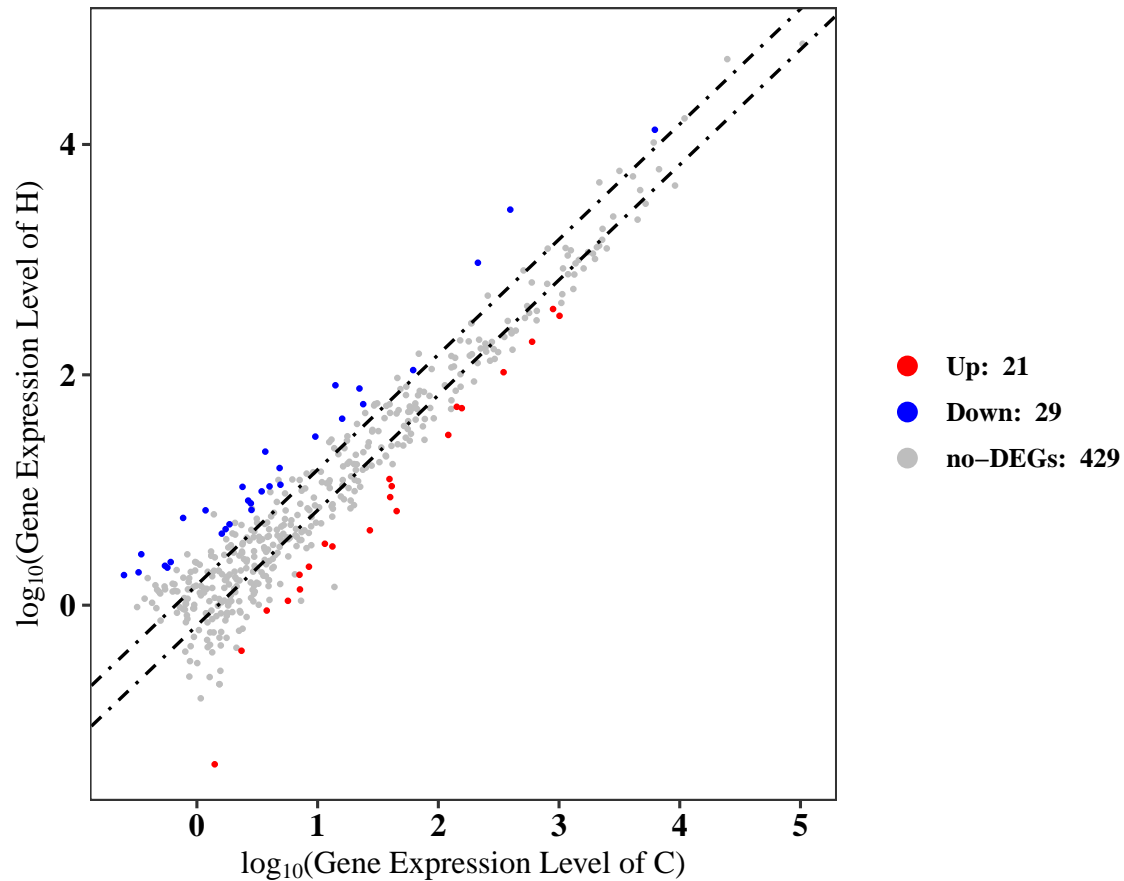

Supplement: Supplemental Information 20 [file peerj-12-16660-s020.zip › Sequence Data/DEG/C_VS_H/Scatter-plot.pdf]

Volcano plot of DEG

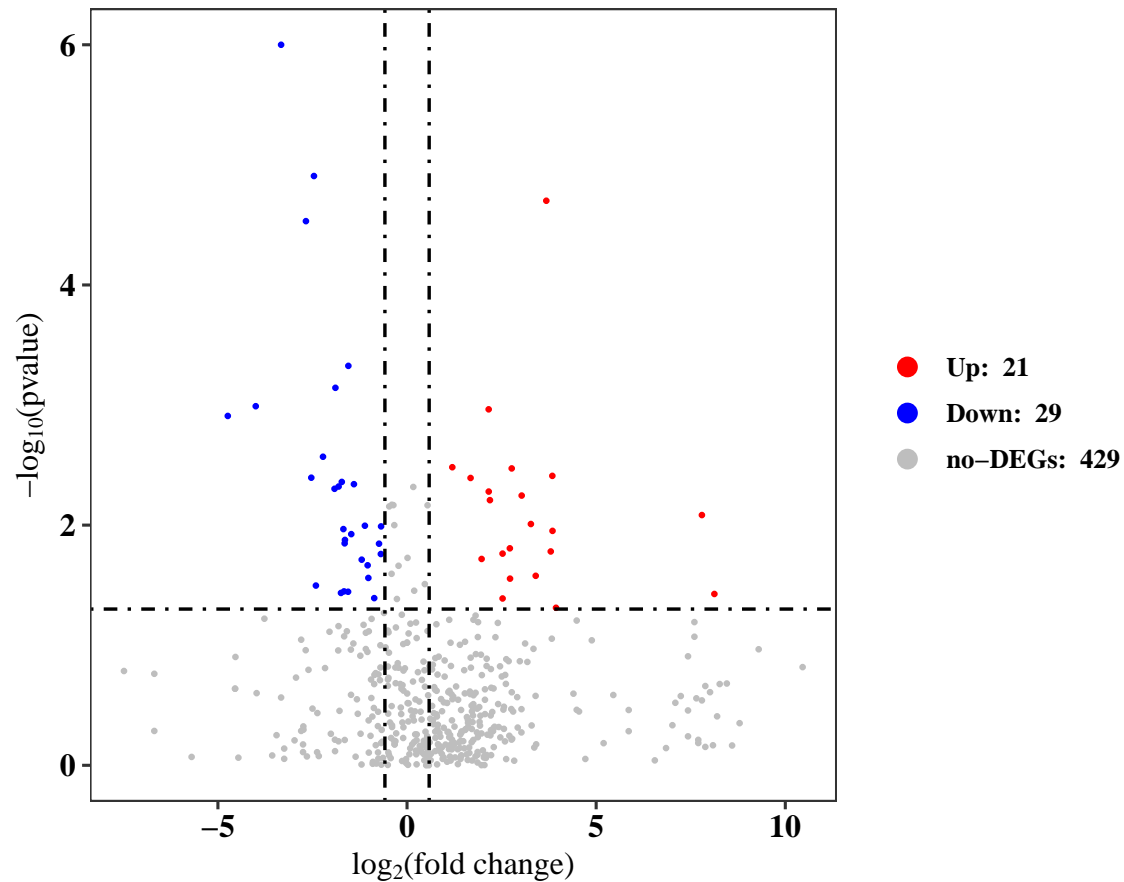

Supplement: Supplemental Information 20 [file peerj-12-16660-s020.zip › Sequence Data/DEG/C_VS_H/volcano-plot.pdf]

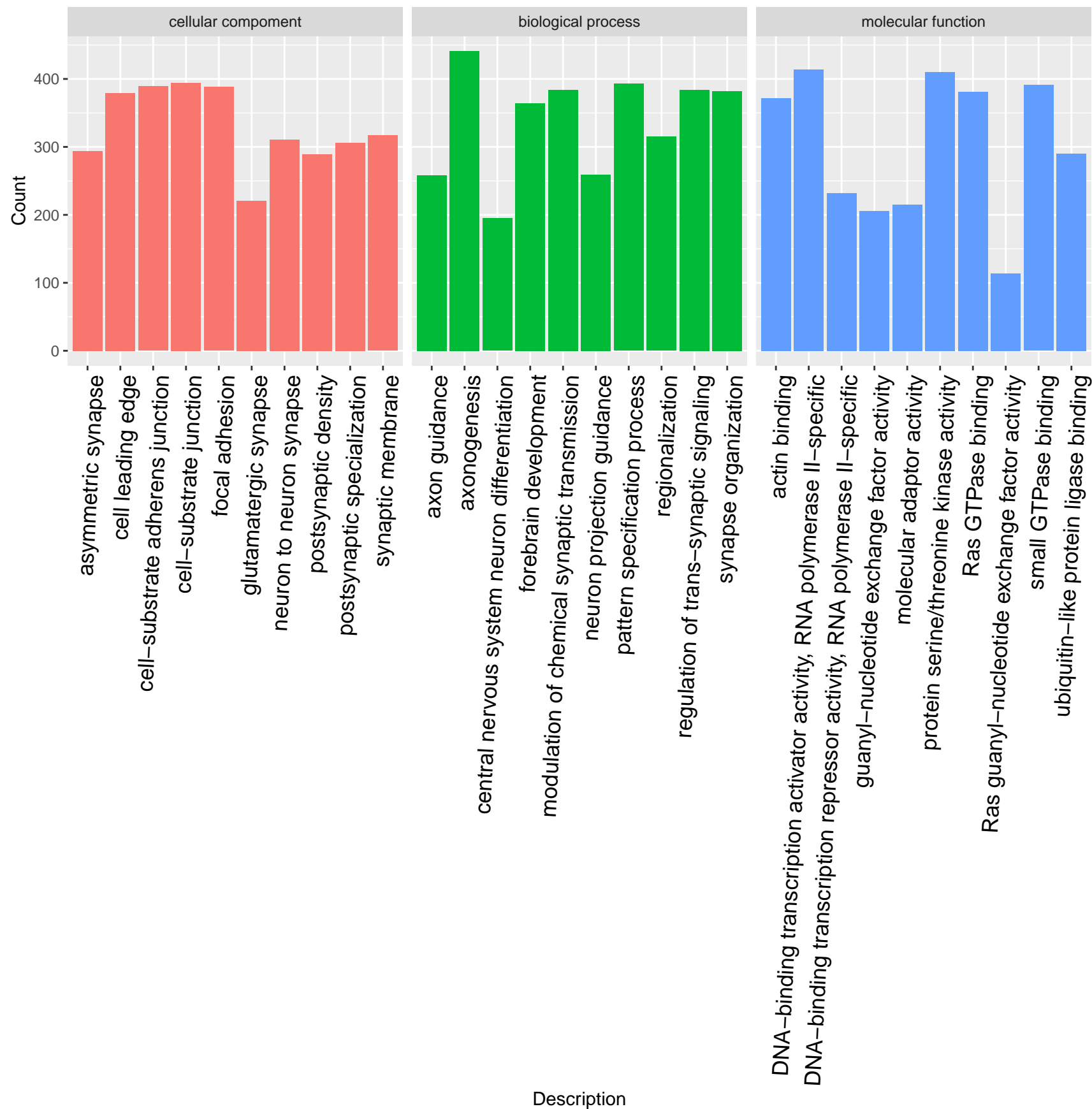

Supplement: Supplemental Information 20 [file peerj-12-16660-s020.zip › Sequence Data/Function/predict/C_VS_H/GO_barplot.pdf]

Description

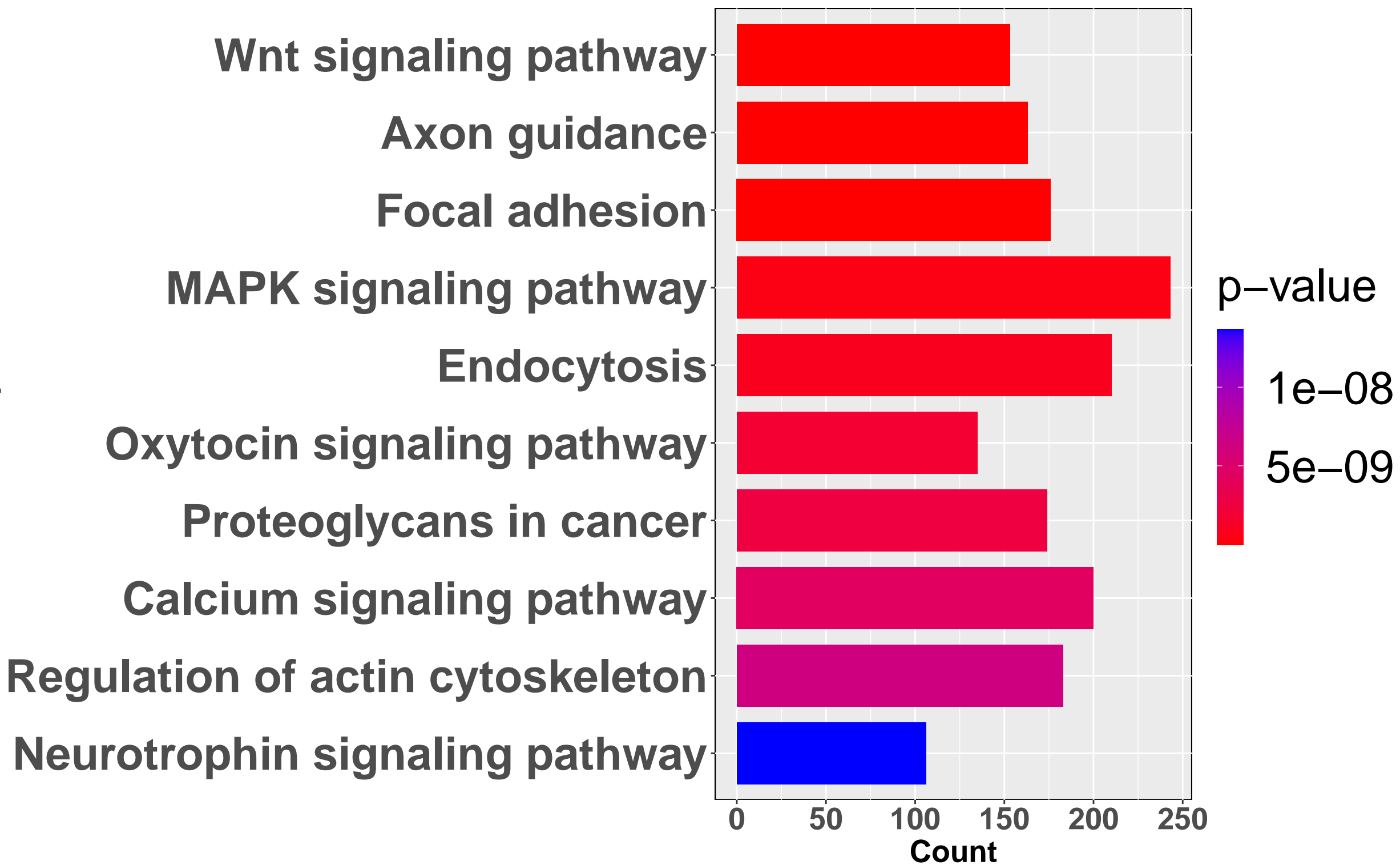

Supplement: Supplemental Information 20 [file peerj-12-16660-s020.zip › Sequence Data/Function/predict/C_VS_H/KEGG_Pathway.barplot.pdf]

Description

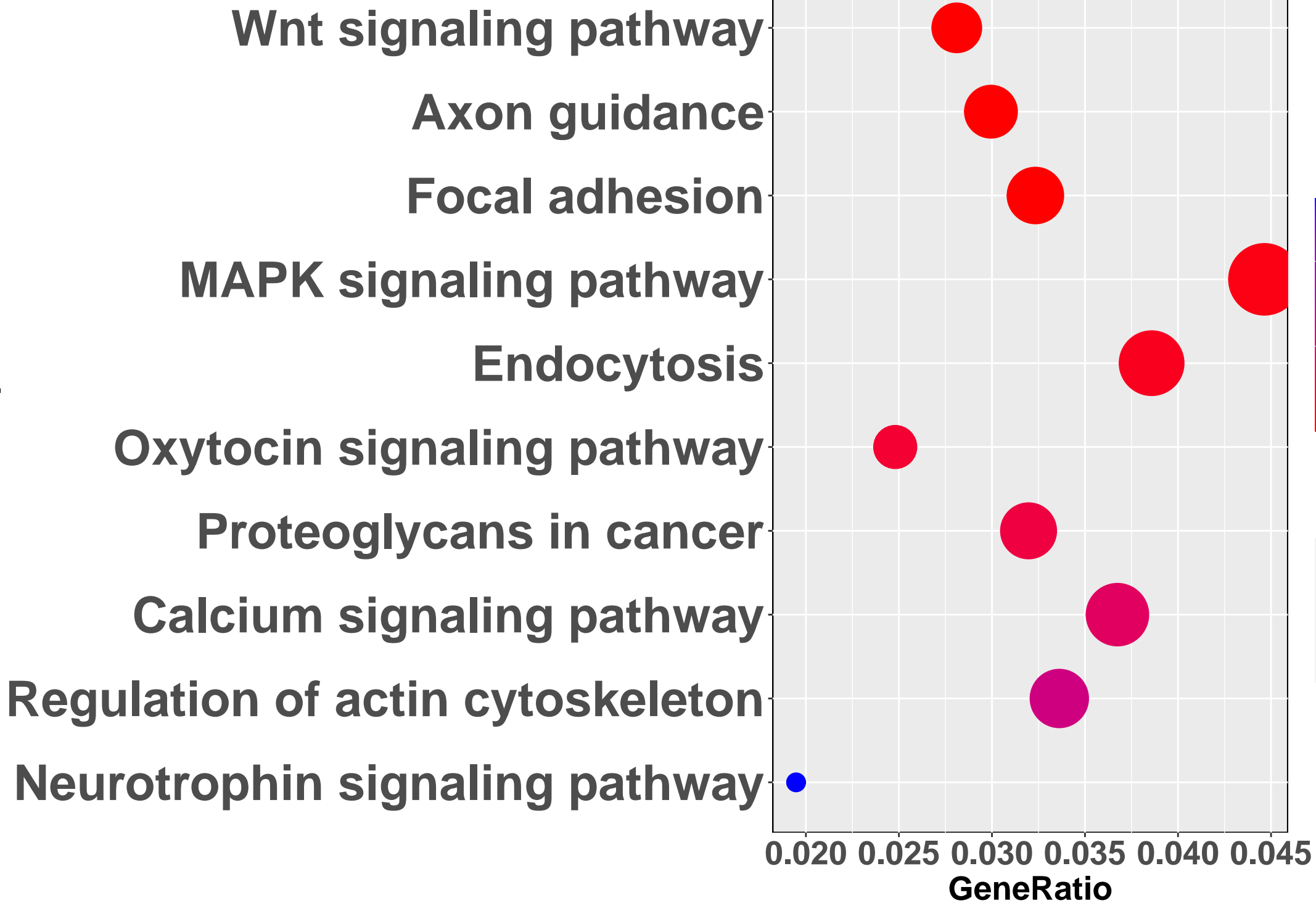

p-value

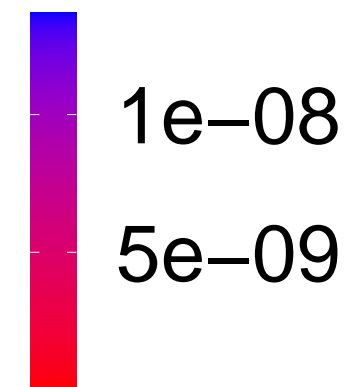

Count

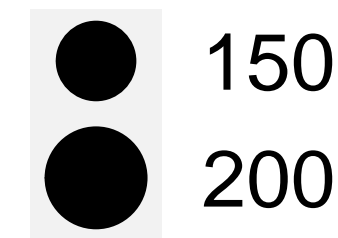

Supplement: Supplemental Information 20 [file peerj-12-16660-s020.zip › Sequence Data/Function/predict/C_VS_H/KEGG_Pathway.dotplot.pdf]

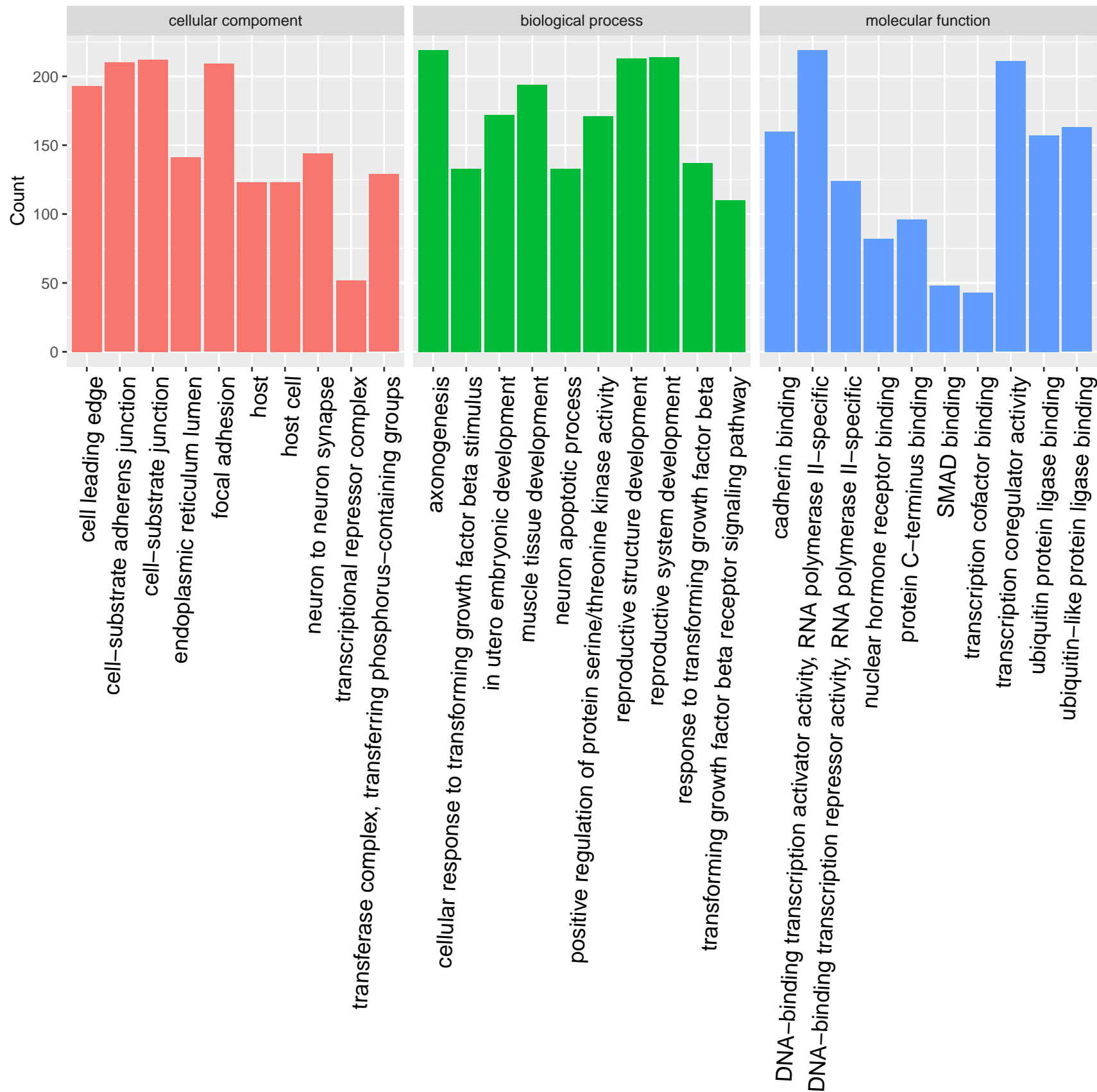

Supplement: Supplemental Information 20 [file peerj-12-16660-s020.zip › Sequence Data/Function/validated/C_VS_H/GO_barplot.pdf]

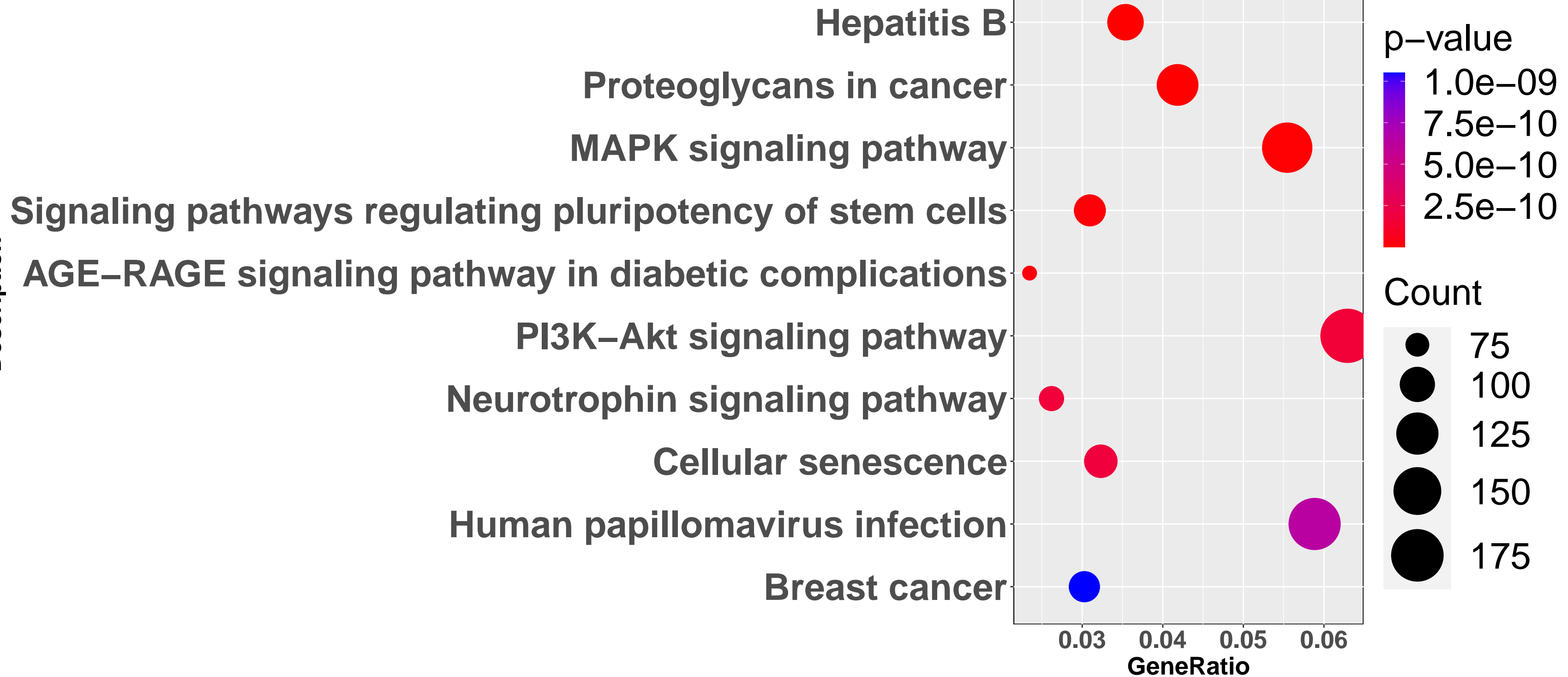

Supplement: Supplemental Information 20 [file peerj-12-16660-s020.zip › Sequence Data/Function/validated/C_VS_H/KEGG_Pathway.dotplot.pdf]

Heatmap

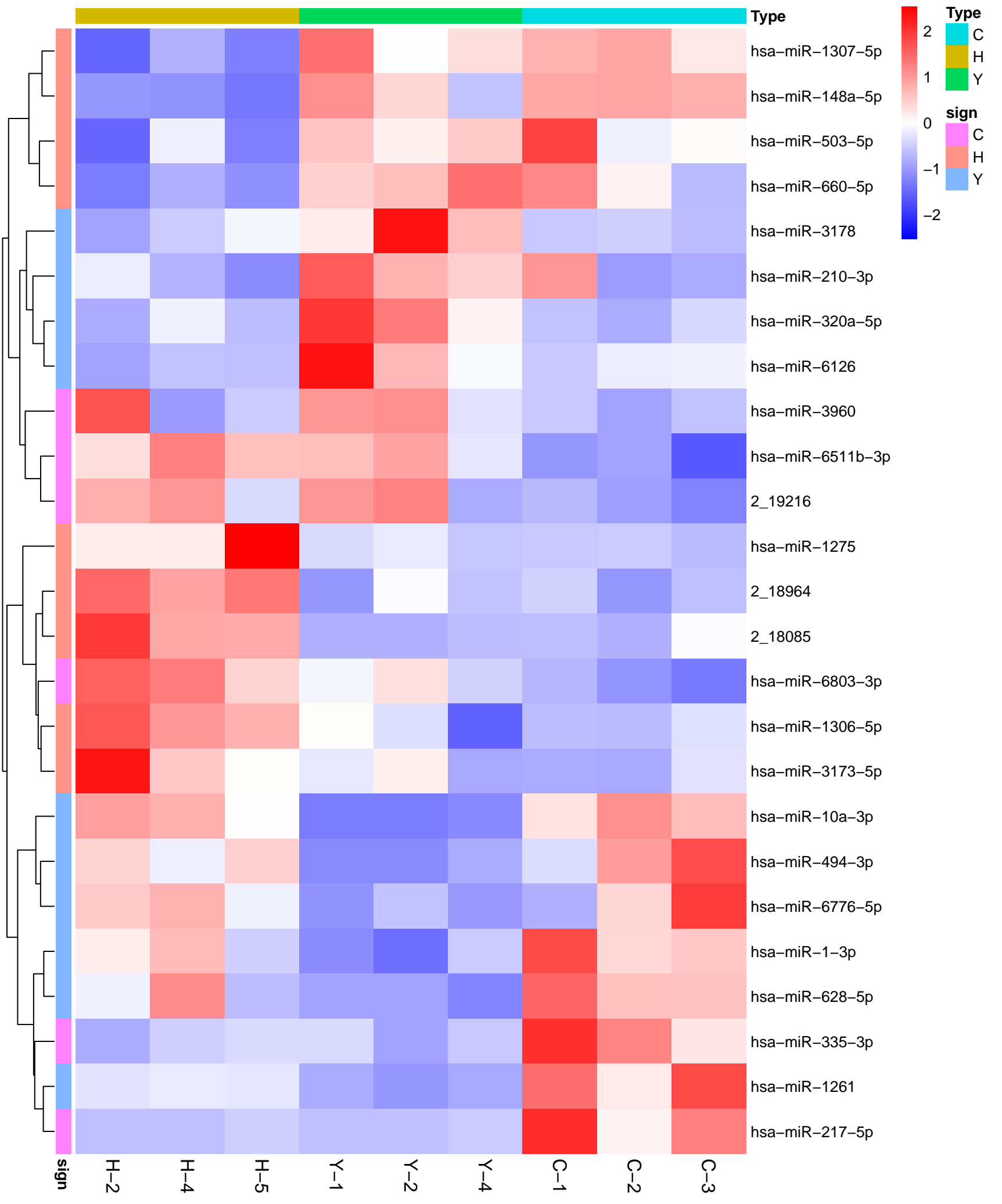

Supplement: Supplemental Information 20 [file peerj-12-16660-s020.zip › Sequence Data/heatmapInclusive.pdf]

Heatmap

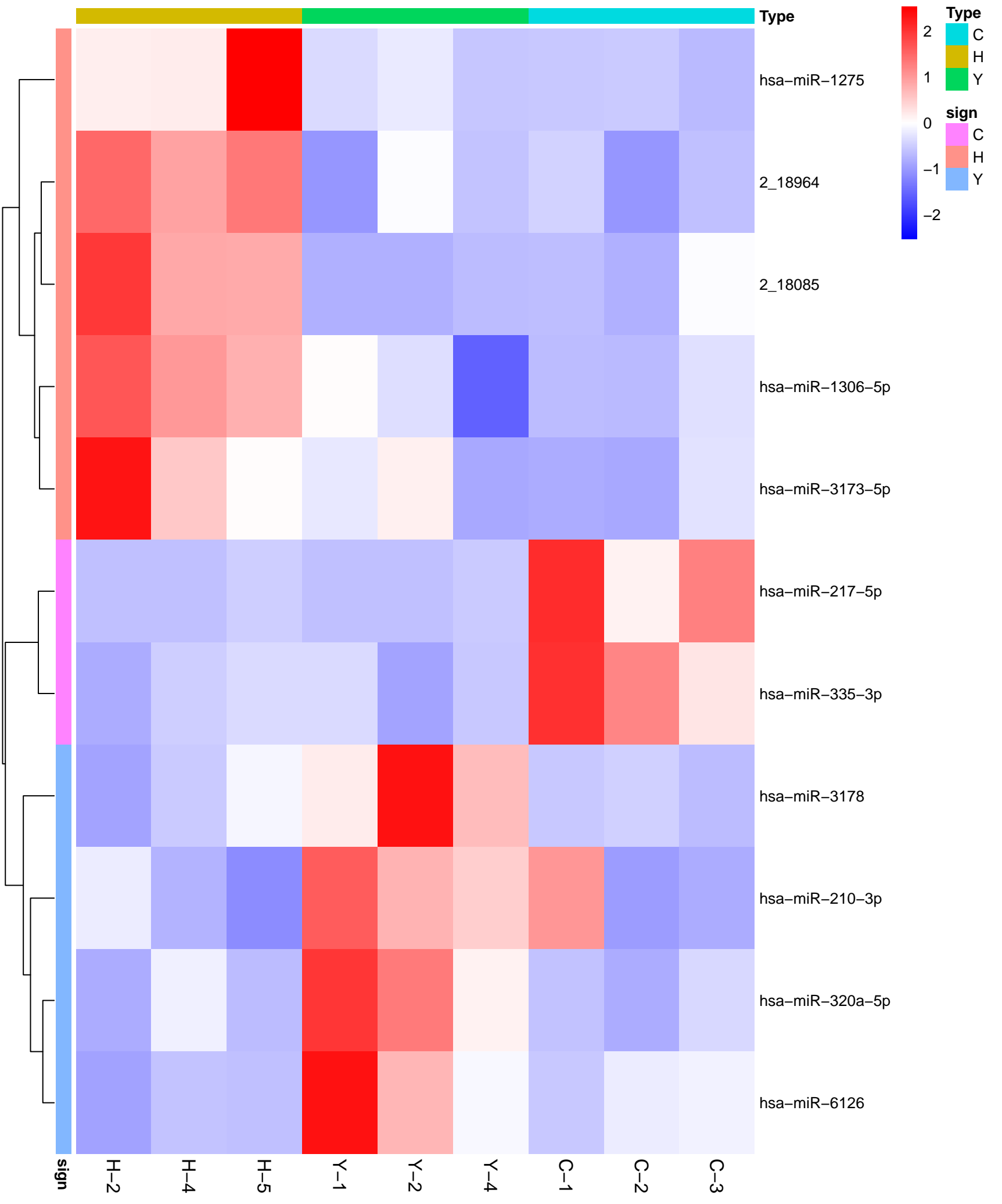

Supplement: Supplemental Information 20 [file peerj-12-16660-s020.zip › Sequence Data/heatmapUnique upregulation.pdf]

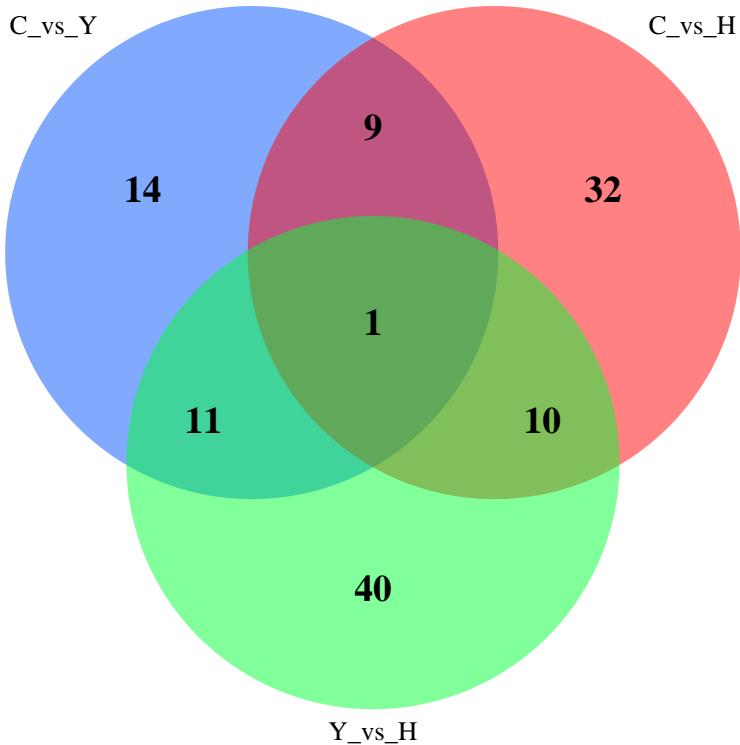

Supplement: Supplemental Information 20 [file peerj-12-16660-s020.zip › Sequence Data/venn.pdf]
